# Molecular Typing of Dengue Virus Circulating in Kolkata, India in 2010

**DOI:** 10.1155/2012/960329

**Published:** 2012-02-13

**Authors:** Arindam Sarkar, Debjani Taraphdar, Shyamalendu Chatterjee

**Affiliations:** ICMR Virus Unit, Kolkata, ID & BG Hospital Campus 57, Dr. S. C. Banerjee Road, Beliaghata, Kolkata 700010, India

## Abstract

Dengue is one of the major public health threats in Kolkata. Every year, blood samples with dengue-like illness are referred to us from different medical colleges and hospitals in Kolkata for the detection of dengue infection in them. In 2010, a total of 378 samples were referred to us for that purpose. All the samples were tested for the detection of IgM antibodies by ELISA method, followed by RT-PCR test for the detection of serotypes. Only 173 samples were ELISA positive. Out of 378 samples, 108 were RT-PCR positive. Out of 108 samples, 74 samples had monotypic infection with different serotypes of DENV and 33 samples had dual infections with DENV-2 and DENV-3. Only one sample had the infection with DENV-1, DENV-2, and DENV-3. DHF was found mainly among the patients, infected with multiple dengue serotypes. Only 3 dengue monotypic infected patients had suffered from DHF.

## 1. Introduction

During the past few decades, dengue fever has gradually become one of the leading causes of morbidity and mortality in tropical and subtropical areas throughout the world [1]. The Dengue virus (DENV), a mosquito-borne member of the family Flaviviridae, circulates as four distinct serological types DENV 1, DENV 2, DENV 3, and DENV 4. Over all, two-fifth of the world population are living in areas, at risk for dengue [2–4]. These four sero types offer cross protection for a very short period. Infection with any of these leads to a mild self-limiting febrile illness (dengue fever, DF). A more severe form of the disease, dengue hemorrhagic fever/dengue shock syndrome (DHF/DSS), is responsible for high mortality rate, especially in children [5]. It has been estimated that about 50 million cases of DF occur annually, with 10,000 infant deaths due to DHF/DSS. DHF/DSS has been postulated to result from immune enhancement after a second heterologous DENV infection [6].

In India, DENV was first isolated in 1946 and many outbreaks have been reported [7–10]. DHF was first reported in Calcutta (Kolkata), West Bengal in 1963 [11], again in 1964 [12]. Since then, there are numerous studies from the Indian subcontinent investigating DHF in various parts of the country [13–22]. But there are no studies investigating the overall prevalence of the dengue serotype circulating in the endemic zone, apart from the epidemic outbreak. The purpose of this paper is to present a comprehensive report on the diagnosis of dengue infection amongst the febrile cases, available from January to December, 2010, in the city of Kolkata and also to identify the serotype presently circulating in this region.

The incidence of DF in the rural West Bengal is on the increase and is spreading to geographic regions not previously affected [23, 24]. It is widely known that dengue is endemic in Kolkata. The city has experienced several dengue episodes in the past centuries [25]. Antibodies against Group B-arthropod-borne viruses in more than 80% of the Kolkata population have been recorded almost fifty years back, and that is too possibly due to the infection by dengue viruses [26].

The present study aimed to identify the serotypes of DENV in the population of Kolkata as well as to study the sociodemographic status in relation to DENV infection.

## 2. Materials and Methods

### 2.1. Study Area

Kolkata is one of the biggest metropolitan cities in India. The present population of Kolkata is 44,86,679 of which 23,62,662 are males and 21,24,017 are females [27]. The city has an international sea and airport and one rail way station (Sealdah) which is busiest in the world. The rail road of this station covers a number of districts, situated at the border of Bangladesh. Adjacent to the city, there is a thickly populated town, Howrah, which has one of the biggest terminating railway stations of the Eastern India. These two rail stations are the gateway of this city. The monsoon begins in June and persists up to the end of October.

### 2.2. Patients and Clinical Specimens

Cases were mainly referred from outpatient department (OPD) and indoor of I.D & B. G Hospital, attached to this unit, from different medical colleges as well as other hospitals in Kolkata along with a short history of the patients. A good number of cases were referred to us by the private practitioners also. In the matter of selection of dengue fever (DF) cases, the following criteria were initially considered: (1) high fever; (2) head ache; (3) retro-orbital pain; (4) nausea/vomiting; (5) malaise/joint pain; (6) generalized skin rashes [28]. In the present study two or more of these criteria, apart from fever, were fulfilled. The possibilities of bacterial and prokaryotic etiology in the collected samples were excluded through investigations at the respective hospitals.

The case history and the investigations of the patients were compiled. In the case of DHF, the history of illness was revealed by the sudden rise of high fever (38.3°C–39.4°C), headache, retro-orbital pain, conjunctival congestion, and facial flashing. Fever sustained for 2–15 days. In addition to that, some cases had the history of hemorrhagic manifestation either with petechiae or with gum bleeding or malena. In such cases, intermittent or biphasic course of fever was recorded, where the first phase of fever persisted for 2–7 days and the second bout of fever persisted for 2–3 days. No cases with the history of plasma leakage were observed. In the admitted cases, we found that fever was also accompanied by generalized malaise and lumbosacral pain. Pulse rate was slow (60–70/min). Those patients represented rashes, appeared on 2–5 days of illness in limbs, sparing palm, and souls. They were nonpruritic in nature and lasted 2–7 days. The hematological examination revealed generalized leucopenia, and platelet count was ≤10^5^/cu mm. All the cases were nondiabetic and had no other physiological complications. Liver was either nonpalpable or just palpable.

All the samples were transported on dry ice to the ICMR Virus Unit. Sera were separated from the collected blood samples, were stored at −80°C, and were tested within 1 month from the date of collection. A total of 378 samples were thus received from the suspected cases and analyzed for the detection of dengue viruses, if any.

### 2.3. Serology

To study the sociodemographic status in relation to DENV infection, all the 378 samples were screened for the presence of dengue IgM antibodies by IgM capture enzyme-linked immunosorbent assay (ELISA): using a kit, prepared by the National Institute of virology, Pune, India, following the prescribed protocol [29]. Optical density (OD) was measured at 492 nm using an ELISA reader (Titertek Multiskan Plus, Lab systems Finland, Type-314).

### 2.4. RNA Extraction

To study the molecular typing of DENV, attempts were made to isolate the RNA from all the samples as well as from four different DENV serotype strains, which were used as positive control. Viral RNA was isolated by using Qiagen viral RNA isolation kit (Qiagen, GmbH, Hilden, Germany) according to the manufacturer's protocol.

### 2.5. RT-PCR

In this study, published primers by Lanciotti et al. were used [30]. In a single tube, viral RNA was converted to a DNA copy (cDNA) prior to enzymatic DNA amplification by the use of reverse transcriptase (RT) and the DENV downstream consensus primer D2-5′-TTGCACCAACAGTCAATGTCTTCAGGTTC-3′ homologous to the genomic RNA of the four serotypes. Subsequent Taq polymerase amplification was performed on the resulting cDNA with the upstream dengue virus consensus primer D1-5′-TCAATATGCTGAAACGCGCGAGAAACCG-3′. Target RNA was amplified in 25 *μ*L volumes containing the following components: 800 mM deoxynucleotide triphosphates (dNTPs), 8 mM dithiothreitol, 0.24 *μ*M each of primers D1 and D2, 0.5 U of AMV RT (Promega, Madison, WI, USA), and 0.625 U of Dreamtaq DNA polymerase (Fermentas Inc., USA). The reactions were allowed to proceed for 1 h at 42°C and then to proceed with 95°C for 3 minutes for initial denaturation followed by 35 cycles of denaturation (95°C for 30 sec), primer annealing (55°C for 1 min), and primer extension (72°C for 2 min) along with final extension (72°C for 5 min).

DENV serotyping was conducted by second-round amplification (nested PCR) initiated with 10 u of diluted material (1 : 100 in sterile distilled water) from the initial amplification reaction. The total 20 *μ*L of reaction mixture was prepared using 2 *μ*L of diluted first PCR products, 0.8 mM dNTPs, 0.5 U of Dreamtaq DNA Polymerase and 0.3 *μ*M of primer D1 and 0.3 *μ*M of dengue virus type-specific primers: TS1 5′-CGTCTCAGTGATCCGGGGG-3′, TS2 5′-CGCCACAAGGGCCATGAACAG-3′, TS3 5′-TAACATCATCATGAGACAGAGC-3′, and TS4 5′-CTCTGTTGTCTTAAACAAGAGA-3′. Dithiothreitol and AMV RT were eliminated. The samples were subjected to initial denaturation (95°C for 3 min) followed by 20 cycles of denaturation (95°C for 30 s), primer annealing (55°C for 1 min), and primer extension (72°C for 1 min) along with final extension (72°C for 5 min). The PCR products were analyzed by running a 1.5% agarose gel stained with ethidium bromide.

## 3. Results

### 3.1. Serology

**Figure 1:**
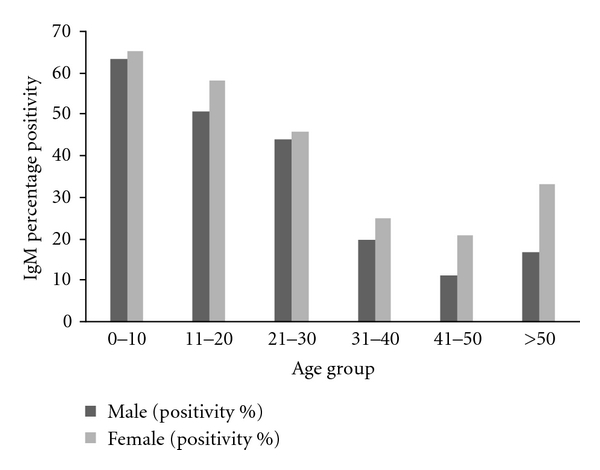
Age and sex wise distribution of dengue IgM-positive cases in 2010.

**Figure 2:**
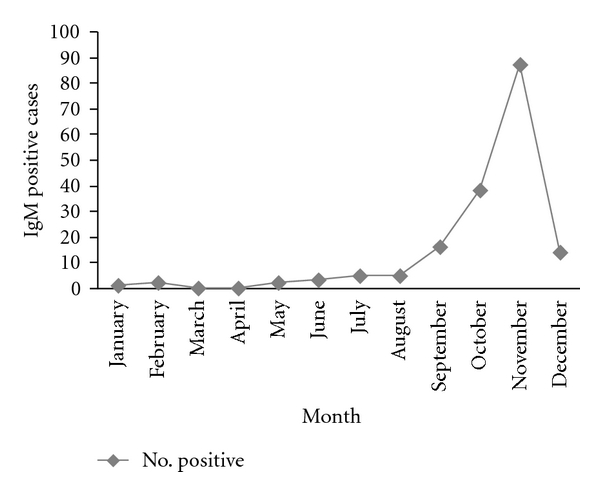
Monthly distributions of IgM-positive dengue cases in 2010.

Out of 378 samples collected, only 173 samples were reactive to dengue IgM antibody by ELISA method. Maximum numbers of IgM positive cases were observed in the age group of 0–10 years in both male and female patients (Figure 1). Females were more affected (46.5%) than the males (45.1%). As regards the seasonal prevalence, it is evident from the result that although sporadic cases obtained throughout the year,dengue cases started from the month of july and attained maximum number of cases in the month of november (Figure 2).

### 3.2. PCR Result

**Figure 3:**
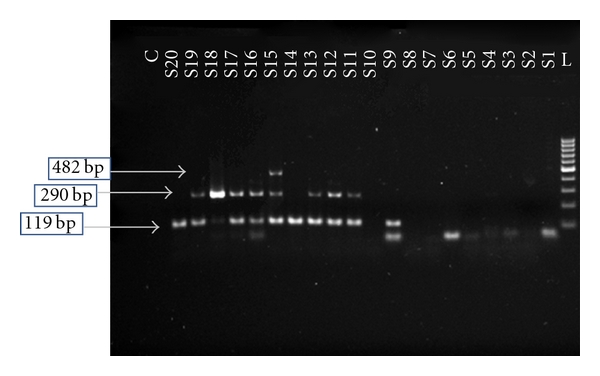
Results of dengue-specific RT-PCR followed by second-round nested PCR of RNA samples, showing band in 1.5% agarose gel electrophoresis stained with ethidium bromide. Lane L: 100 bp DNA ladder, Lane S1–S8 and S10: negative sample, Lane S9, S14 and S20: sample positive for DEN-2 (119 bp), Lane S11–S13 and S16–S19: sample with dual infection of DEN-2 (119 bp) and DEN-3 (290 bp), Lane S15: sample with concurrent infection of DEN-1 (482 bp), DEN-2 (119 bp), and DEN-3 (290 bp), and Lane C: negative control.

Out of 378 samples, 108 were RT-PCR positive. Seventy-four samples (68.5%) had the DENV infection by single different serotypes, of which 7 samples (9.5%) had the monotypic infection with DENV-1, 45 samples (60.8%) had the monotypic infection with DENV-2 and 22 samples (29.7%) had the monotypic infections with DENV-3 serotype. Both DENV-2 and DENV-3 serotypes were detected in 33 samples (30.6%). Only one sample had all the three serotypes, that is, DENV-1, DENV-2, and DENV-3. No DENV-4 serotype was detected in those samples. Out of 22 DHF cases, 3 were found with single-serotype infection and 18 cases with dual infections of DENV 2 and DENV 3. Only a female patient of 24 years of age had the infection with DENV-1, DENV-2, and DENV-3 serotypes at a time and also suffered from DHF. Clinical data regarding the death of the DHF cases was inadequate to reach any conclusion about the severity of illness. The specimen containing DENV-1, DENV-2, and/or DENV-3 is identified by the detection of a DNA band of 482, 119, or 290 bp in size, respectively, on 1.5% agarose gel, loaded with nested PCR products along with positive controls, stained with ethidium bromide (Figure 3).

## 4. Discussion

The monitoring of DENV activity is required for public health importance, as the dengue fever and DHF/DSS are increasing worldwide and are spreading in the places, where it was previously not reported. The first isolation of DENV serotype 1 and 4 was reported from India in 1964 [31, 32] and serotype 3 in 1968 [18]. Although concurrent infection with more than one serotype of DENV in the same individual is uncommon, high percentage of concurrent infections with different DENV serotypes had been detected at an outbreak in Delhi, India, in 2006 [33]. Kolkata is a dengue endemic zone; frequent outbreaks of DF and DHF have been occurring since last centuries. In Kolkata, dengue was first documented in 1824 and several epidemics took place in the city during the years 1836, 1906, 1911, and 1972, affecting 40% of the city people [34]. The last large-scale dengue outbreak has been recorded in the year 2005 [35]. In the rural areas of West Bengal, dengue is gradually spreading and establishes new reports [23, 24]. No continuous monitoring of the molecular detection of the dengue serotype has yet been attempted in the city of Kolkata, either in epidemic or in sporadic dengue outbreaks.

Out of 378 blood samples, initially 173 (45.76%) cases were reactive to dengue IgM antibody. Agewise distribution revealed that the highest number of dengue cases were detected in the age group of 0–10 years, followed by 11–20 years and above (Figure 1). In the highest age group (above 50 years), the number of positive cases were too small and only 16.6% dengue IgM positivity was found in the male individuals. In all the age groups, females were more affected than the males (Figure 1). As the vector mosquitoes (*Aedes *sp.) are domestic and peridomestics in nature, the females get more exposure than the males, as most of the time they reside inside the house. During the year-round study, although small number of samples from suspected dengue cases were referred to us during the period from January to May, only a few were positive to dengue IgM antibody by ELISA method. It is evident from our study (Figure 2) that the dengue cases actually started from the month of June and attained its peak in the month of November during this year, which is the post-monsoon period. It may be explained by the fact that the stagnant fresh water during the rainy seasons (June to October) favoured the breeding of the vector mosquitoes.

For the molecular detection, RT-PCR was performed with all samples, of which 108 samples were positive by that method. Forty-one samples were both ELISA and RT PCR positive. A total of 74 samples had the monotypic infections, involving DENV 1, DENV 2, and DENV 3, of which DENV 2 was predominated. All these were IgM negative. Conversely, 33 samples had the dual infections with DENV 2 and DENV 3, of which all the samples were IgM reactive. Only one sample produced prominent band against DENV1, DENV 2, and DENV 3 and also contained IgM antibody. The detection of the viral RNA in presence of the IgM antibody may be explained by the fact that, due to the consecutive infection, the IgM detected in those samples possibly appeared due to the initial infection, which is evident by the intensity of the bands in gel electrophoresis (Figure 3). The hemorrhagic manifestation was found mainly among the patients, infected with multiple dengue serotypes. Only 3 dengue monotypic infected patients had suffered from DHF. DHF cases were observed mainly among the young and young-adult age groups (0–30 years) which might be due to the absence of immunity against all serotypes of DENV in them.

Although the increasing trend of cocirculation of multiple DENV serotypes suggests that Kolkata is becoming a hyperendemic state, a large-scale monitoring on the circulating strains of DENV is highly required to draw a definite conclusion.
